# Brain Entropy Mapping in Healthy Aging and Alzheimer’s Disease

**DOI:** 10.3389/fnagi.2020.596122

**Published:** 2020-11-10

**Authors:** Ze Wang, for the Alzheimer’s Disease Neuroimaging Initiative

**Affiliations:** Department of Diagnostic Radiology and Nuclear Medicine, University of Maryland School of Medicine, Baltimore, United States

**Keywords:** resting state fMRI, entropy, pathology, reserve, AD, MCI

## Abstract

Alzheimer’s disease (AD) is a progressive neurodegenerative disease, for which aging remains the major risk factor. Aging is under a consistent pressure of increasing brain entropy (BEN) due to the progressive brain deteriorations. Noticeably, the brain constantly consumes a large amount of energy to maintain its functional integrity, likely creating or maintaining a big “reserve” to counteract the high entropy. Malfunctions of this latent reserve may indicate a critical point of disease progression. The purpose of this study was to characterize BEN in aging and AD and to test an inverse-U-shape BEN model: BEN increases with age and AD pathology in normal aging but decreases in the AD continuum. BEN was measured with resting state fMRI and compared across aging and the AD continuum. Associations of BEN with age, education, clinical symptoms, and pathology were examined by multiple regression. The analysis results highlighted resting BEN in the default mode network, medial temporal lobe, and prefrontal cortex and showed that: (1) BEN increased with age and pathological deposition in normal aging but decreased with age and pathological deposition in the AD continuum; (2) AD showed catastrophic BEN reduction, which was related to more severe cognitive impairment and daily function disability; and (3) BEN decreased with education years in normal aging, but not in the AD continuum. BEN evolution follows an inverse-U trajectory when AD progresses from normal aging to AD dementia. Education is beneficial for suppressing the entropy increase potency in normal aging.

## Introduction

Alzheimer’s disease (AD) is a neurodegenerative disease that has impacted millions of elderly people but still remains incurable (Ferri et al., 2005; Reitz and Mayeux, 2014). Although AD has been well characterized by AD pathology and clinical symptoms, a major barrier to research progress is the unclear mechanism for how and when normal aging progresses into AD dementia (Kumar and Singh, 2015; Mehta and Yeo, 2017) and why AD symptoms often emerge many years later than AD pathology. This pathology vs. symptom discrepancy (Jack et al., 2010; Jack and Holtzman, 2013) suggests that there may exist a reserve of brain function according to the seminal “cognitive reserve” (CR; Stern, 2006; Stern et al., 2018) model. This reserve may compensate brain damage–induced functional abnormalities in normal aging but fails to do that after disease conversion. To characterize the brain function reserve, we need a more tangible proxy. One candidate is the resting-state brain activity which matches the latent function reserve in two perspectives: first, it is an ongoing process non-specific to any overt brain function; second, it has been postulated to play a role in brain function facilitation (Raichle et al., 2001; Raichle and Gusnard, 2002; Raichle, 2011). Resting-state fMRI (rsfMRI) represents the most widely used tool for studying resting brain activity and has been used to assess neural correlates of brain reserve through the inter-regional functional connectivity (FC) analysis (Arenaza-Urquijo et al., 2013; Bozzali et al., 2015; Marques et al., 2016; Franzmeier et al., 2017; Li et al., 2020). An overall picture revealed by these studies is that higher CR measures are related to stronger FC in distributed brain regions including the default mode network (DMN) area and weaker FC in other restricted focal regions. Because FC is defined by the inter-regional signal correlation in the seed-based FC (Biswal et al., 1995) or the associations to a common temporal fluctuation pattern in the spatial independent component decomposition (Calhoun et al., 2001; Hyvärinen et al., 2001; Beckmann and Smith, 2004), it does not tell anything specific to regional brain activity.

**Figure 1:**
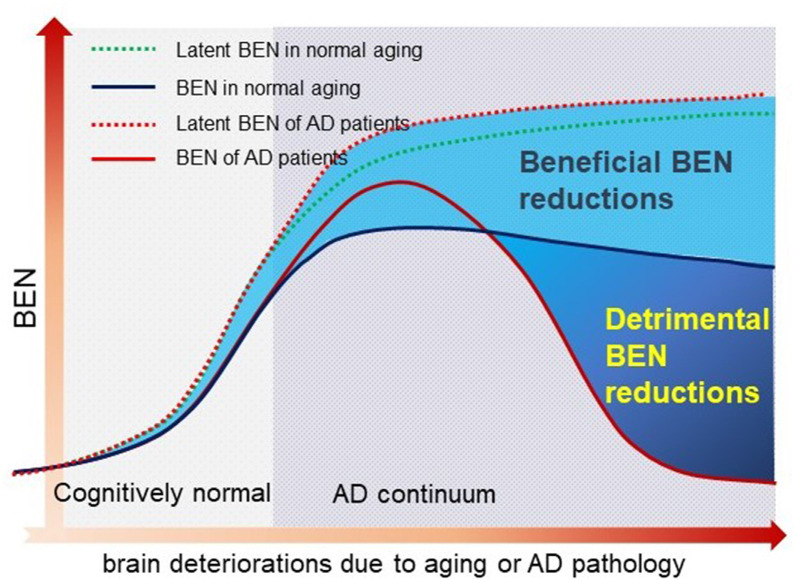
A hypothetical brain entropy (BEN) model for normal aging and Alzheimer’s disease (AD). The dotted line shows the latent BEN evolution trend as a result of the aging-related accumulated brain deteriorations. The dashed line represents the actual BEN evolution curve after brain reserve compensation which imposes negative entropy brings down the total BEN. Catastrophic BEN reduction may start at the disease conversion time due to a potential overaction of the brain reserve.

In this study, we proposed entropy of each local voxel as a regional proxy of brain reserve. Entropy is a physical measure for a dynamic system with high entropy indicating less order and more irregularity. It may be informative for delineating the aforementioned AD pathology vs. symptom discrepancy because aging is known to have progressive brain deteriorations (Hayflick, 2004; Drachman, 2006) which inevitably increase the brain entropy. High entropy corresponds to low temporal coherence, which is detrimental to brain functional organization and has to be counteracted to keep the normal brain functionality. Because brain reserve is defined by brain function facilitation and compensation, assessing entropy of functional brain activity may provide a direct outcome measure of the latent brain reserve. In a pilot study (Wang, 2020a,b; full article under separate review) based on data from 862 healthy adults from the human connectome project (Van Essen et al., 2013), we found that brain entropy (BEN) in the DMN (including precuneus, bilateral parietal cortex, and part of temporal cortex) and the executive control network (ECN; including the dorsolateral prefrontal cortex and lateral parietal cortex) increases with age but decreases with education years (an indicator of cognitive reserve for compensating brain dysfunctions) and that lower BEN in DMN and ECN is associated with better performance of cognitive functions. These data suggest the feasibility of BEN for characterizing the latent brain reserve compensation outcome. Although the compensation may be sufficient in normal aging, they may become insufficient when disease progresses, which can reciprocally trigger reserve overactions, leading to a catastrophic reduction of BEN as found in previous biophysiological recording–based AD entropy studies (Stam et al., 2003; Jeong, 2004; Abásolo et al., 2006; Gómez and Hornero, 2010; Mizuno et al., 2010; Yang et al., 2013). To explain this apparent opposed entropy change pattern in normal aging and AD, we proposed a heuristic BEN model as shown in Figure 1. This model considers low BEN in DMN and ECN as beneficial for normal aging because low brain entropy corresponds to high temporal coherence which is evidenced to be important for brain function (Buzsáki and Draguhn, 2004; Buzsaki, 2006; Schroeder and Lakatos, 2009; Saleh et al., 2010; Buzsáki and Watson, 2012; Henry and Obleser, 2012; Lega et al., 2012; Thut et al., 2012; Calderone et al., 2014; Reinhart and Nguyen, 2019). However, in AD, our model predicts a detrimental large BEN reduction in DMN/ECN, indicating a failure of the functional compensation role of brain reserve in AD (Stern, 2006, 2012; Stern et al., 2018). The accumulating brain errors or deteriorations will increase BEN and the risk of brain dysfunction if no compensations occur. This potency, however, can be substantially counteracted by brain reserve or other compensatory mechanisms, resulting in a slowly increasing and then flat topping BEN evolution curve in normal aging (the dashed blue line in Figure 1). When the BEN increase latency reaches a critical point where brain dysfunction cannot be fully compensated anymore, reserve overaction may be triggered, leading to an apparent BEN reduction (the red solid curve in Figure 1). When disease progresses, BEN reduction may be accelerated further by other detrimental factors such as the accumulation of Aβ deposition and perfusion deficits. Both Aβ decomposition and hypoperfusion may cause or be associated with BEN reductions through the CBF vs. brain coherence associations: lower CBF correlates with higher brain activity coherence (Sharbrough et al., 1973; Foreman and Claassen, 2012; higher coherence corresponds to lower BEN).

The main purpose of this study was to assess the feasibility of BEN as an outcome measure of the latent brain function reserve and to evaluate the hypothetical BEN model by leveraging the relatively large data from the AD Neuroimaging Initiative (ADNI)^1^ and our recently developed rsfMRI-based BEN mapping tool (Wang et al., 2014). The model was assessed using the cross-sectional ADNI rsfMRI data. We hypothesized that AD patients have lower BEN than cognitively healthy elderlies; BEN increases with age in normal aging but not in AD. The association of BEN to function reserve was examined through the correlation between BEN and education, cognitive function measures, and AD pathology measures. Education is a main contributing factor of cognitive reserve (Stern et al., 2018). Longer education years have been demonstrated to be beneficial for combating cognitive impairments. In accordance with the BEN model, we hypothesized that longer education years are associated with reduced BEN in normal aging but not in AD. The entire study reported in this paper is a full expansion of a small sample-based preliminary study (Li and Wang, 2016).

## Materials and Methods

### Human Subjects

All human subjects’ data included in this study were downloaded from the ADNI database^1^. Reanalysis of ADNI data was approved by institutional review boards of all participating institutions and written informed consent was obtained from all participants or authorized representatives. Subjects were limited to those with rsfMRI data acquired with the traditional gradient-echo-weighted echo-planar imaging sequence by May 2018. Full inclusion and exclusion criteria for ADNI are described at www.adni-info.org. In brief, patients with mild cognitive impairment (MCI) were classified essentially in the manner described by Petersen (2004), but were then further divided into “early” and “late” groups (i.e., EMCI and LMCI, respectively) based on performance on the Wechsler Memory Scale–Revised Logical Memory II (WMS-LM). The EMCI group was defined based on scores between the cutoff of normal and that of the LMCI group. A total of 211 subjects whose rsfMRI data met all QC criteria were analyzed. Detailed characteristic information and the number of subjects in each sub-group are listed in Table 1.

**Table 1:** Human subject characteristics.

Diagnosis group	HC	SMC	EMCI	LMCI	Alzheimer’s disease	*P*-value
Number	54	27	58	38	34	–
Gender (M/F)	24/28	12/15	22/35	24/14	16/18	0.253
Age (mean ± SD, range)	75.3 ± 6.96, 65–95	72.44 ± 5.49, 65–83	71.53 ± 6.93, 56–89	71.89 ± 8.26, 57–88	72.47 ± 7.06, 56–87	0.082
*APOE* ε4 allele	30.00%	29.63%	45.61%	35.89%	70.59%	**1.9E−03**
MMSE (mean ± SD)	27.56 ± 5.71	28.78 ± 1.48	25.93 ± 7.94	26.59 ± 6.12	22.21 ± 4.47	**1.59E−4**

**P*-values were assessed due to significant differences among diagnosis groups and were computed using one-way ANOVA (except for gender using χ^2^ test). HC, healthy control; SMC, significant memory concern; EMCI, early mild cognitive impairment; LMCI, late mild cognitive impairment; MMSE, Mini-Mental State Examination; values in bold signify *p* < 0.05*.

### MRI Data Acquisition

Both high-resolution structural MRI data and rsfMRI data were downloaded from the ADNI website^1^. The structural images were acquired using a 3D magnetization-prepared rapid acquisition with gradient echo T1-weighted sequence with the following parameters: repetition time/echo time/inversion time = 2,300/2.98/900 ms, 176 sagittal slices, within plane field of view = 256 × 240 mm^2^, voxel size = 1.1 × 1.1 × 1.2 mm^3^, flip angle = 9°, bandwidth = 240 Hz/px. rsfMRI was acquired using a gradient echo-weighted echo-planar imaging sequence with the following acquisition parameters: repetition time/echo time = 3,000/30 ms, number of axial slices = 48, slice thickness = 3.3, flip angle = 80°, within plane field of view = 212 × 212 mm^2^, and number of timepoints = 140.

### MRI Data Preprocessing

MR image preprocessing was conducted using the pipeline included in BENtbx (Wang et al., 2014) with the following steps: slice timing correction, motion correction, temporal nuisance correction, spatial smoothing, inter-modality coregistration (structural image and rsfMRI images), and spatial normalization. These procedures were implemented in Matlab m-script. Coregistration and spatial normalization were based on functions provided by SPM (version 12^2^); other steps were based on custom code written by the author. The first two rsfMRI images were excluded to allow rsfMRI signal reach the steady state. Subjects included in the following analyses had no more than 2 mm translational motions and no more than 2° of angular motions. Subjects with mean framewise displacement (Power et al., 2012) greater than 0.5 mm were excluded too. Residual motions were regressed out from the rsfMRI time series in the temporal nuisance correction step. The Diffeomorphic Anatomical Registration Through Exponential Lie Algebra algorithm (Ashburner, 2007) implemented in SPM12 was used to generate a local brain template based on all subjects’ gray matter and white matter probability maps. The template was registered into the Montreal Neurological Institute (MNI) standard space using a linear affine transformation. With these two transforms, each individual subject’s rsfMRI was mapped into the MNI space for group-level analysis. BEN calculation was performed for each voxel of the preprocessed rsfMRI data using two iterative processes. Given the rsfMRI time series of any voxel, a sliding window with a length of *m* consecutive timepoints was used to extract all possible data segments as illustrated by the colored rectangles overlaid on the time series and the associated arrows. For the *i*-th data segment, its Chebyshev distance to another segment was calculated. If the distance was smaller than the cutoff threshold *r*, it was considered as a “match”. *r* and *m* were selected to be *r* = 0.6 and *m* = 3 as evaluated in Wang et al. (2014). The same procedure was iterated until the seed segment was compared with all other segments and the total number of matches was recorded as ${\text{B}}_{\text{i}}^{\text{m}}$(r) and the sum of ${\text{B}}_{\text{i}}^{\text{m}}$ over all segments was recorded by ${\text{B}}_{\text{i}}^{\text{m}}$(r). Next, the sliding window length was increased by 1 to be m + 1. The aforementioned matching process was repeated to get the total number of matches A^(m + 1)^(r) for all segments with a length of m + 1. Following the Sample Entropy formula, entropy was finally calculated as the logarithm of the ratio of B^m^(r)/A^(m + 1)^(r). This process is theoretically equivalent to calculating the negative natural logarithm of the conditional probability that two temporal segments of the entire data time series similar for m points remain similar for m + 1.

### Cerebrospinal Fluid (CSF) Biomarker

The amyloid-β 1–42 peptide (Aβ_1–42_) and total tau (t-tau) measured in the baseline CSF samples were obtained from the ADNI database^1^. Sample acquisition and quality control of CSF were performed as described previously (Shaw et al., 2009). Mean and SD of t-tau/Aβ_1–42_ ratio were calculated, while subjects with greater or smaller than 6 SD from the mean value were regarded as outliers. Only one subject was out of this range and was subsequently excluded from the following analysis.

### Statistical Analysis

An ANOVA model was used to examine BEN difference between controls and patients at different disease stages. Disease diagnosis vs. pathology interactions were modeled. Age, sex, race, and education were included as variables. Cross-sectional BEN difference and age, sex, and education effects were assessed using *ad hoc* contrast analysis as mentioned previously. Disease vs. age, sex, and education interactions on BEN were examined. Voxelwise multiple regressions were used to assess age, sex, and education effects and the associations of BEN to delayed recall (for memory), memory test results in the Rey Auditory Verbal Learning Test (RAVLT; Schmidt, 1996), the total score of Functional Activity Questionnaire (FAQ; Pfeffer et al., 1982; Marshall et al., 2015), and the Mini-Mental State Examination (MMSE). The rationales for choosing these neuropsychological measures are memory dysfunction is a hallmark of clinical AD symptoms and is widely assessed by delayed recall and RAVLT; AD patients present characteristic daily function impairment which can be measured by FAQ; MMSE is the most often used short screening tool for measuring the overall cognitive impairment. Sex, age, and education level were included as nuisance covariates in these regression models. Additional multiple-regression models were used to assess associations of BEN vs. CSF Aβ (Aβ_1–42_) concentration.

### Data Availability

BENtbx used in this study is available from https://www.cfn.upenn.edu/~zewang/BENtbx.php. ADNI data are available from loni.usc.edu/adni. Analysis results are available from the author by request.

## Results

**Figure 2:**
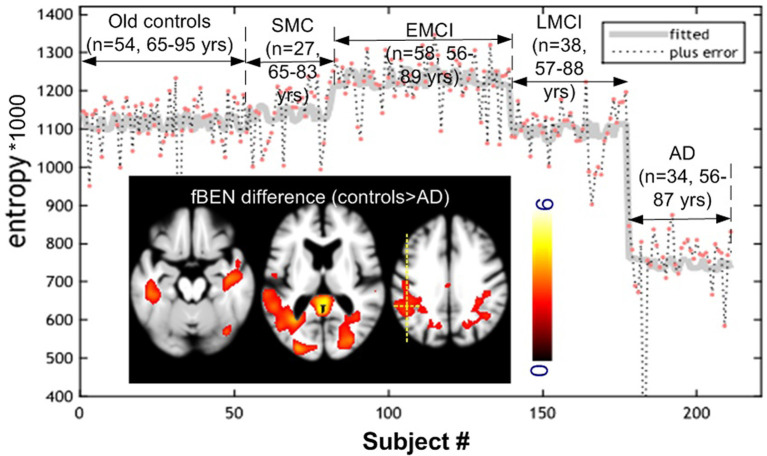
Cross-sectional brain entropy (BEN) profile identified from ADNI rsfMRI. BEN slightly increased from old controls to significant memory concern (SMC) and then early mild cognitive impairment (EMCI), but reduced from EMCI to late mild cognitive impairment (LMCI), and then AD at an accelerated pace. The inset figure shows the post hoc control > AD (red spots in the three image slices) BEN difference (*P* < 0.005, cluster size > 300 corrected). The gray curve was the fitted line of BEN from different populations from the same position marked by the yellow cross. Color bar indicates the display window for the t-map shown in the three image slices.

Age difference was significant only between controls and EMCI (p = 0.02). Figure 2 shows the one-way ANOVA results. BEN was significantly (*F*-test, *p* < 0.05, family-wise error corrected) different within the whole brain among the five populations (elderly controls, significant memory concern (SMC), EMCI, LMCI, and AD). The hot spots overlaid on the three axial image slices in Figure 2 are the *post hoc* voxelwise BEN difference between AD and controls. At *p* < 0.005, cluster size > 300 [AlphaSim (the updated version) corrected], AD showed reduced BEN in MTL including hippocampus (HIPP), inferior temporal cortex, precuneus, and parietal cortex (part of the DMN). No BEN increase was observed across the brain in AD as compared with controls. BEN was extracted from a voxel in left parietal cortex as marked by the yellow dotted cross. Both the scatter plot and the fitted curve demonstrate an inverse-U shaped transition pattern of BEN from cognitively normal elderly controls to AD: BEN slightly increased from controls to SMC, then to EMCI, but quickly dropped to be below BEN of controls in LMCI, and fell further in AD at an accelerated pace. This curve was very similar at different voxels in DMN, PFC, and other brain regions.

**Figure 3:**
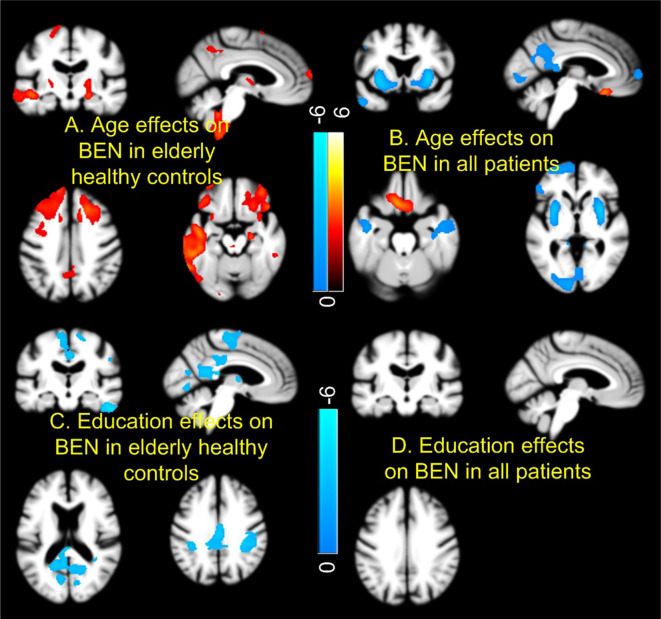
Age and education effects of brain entropy (BEN). Controls and patients [mild cognitive impairment (MCI) and AD] showed opposite BEN vs. age relationship in most part of the brain **(A,B)**. Education years correlated with reduced BEN in default mode network in controls **(C)**, but not in patients **(D)**. Red and cool colors mean positive and negative correlations, respectively. Color bars indicate the display windows of the *t*-maps of the regression analysis.

Figure 3 shows the age and education effects of BEN. Controls showed age-related BEN increase [Figure 3A; *p* < 0.005, cluster size > 200 (corrected using AlphaSim)] in precuneus, MTL, and PFC. Education years were negatively correlated to BEN in controls (Figure 3C). By contrast, the age effects were mostly flipped to the opposite direction (a negative correlation) in the combined patient group (SMC + EMCI + LMCI + AD; Figure 3B) and no education effects were found in the composite patient group at all (Figure 3D).

**Figure 4:**
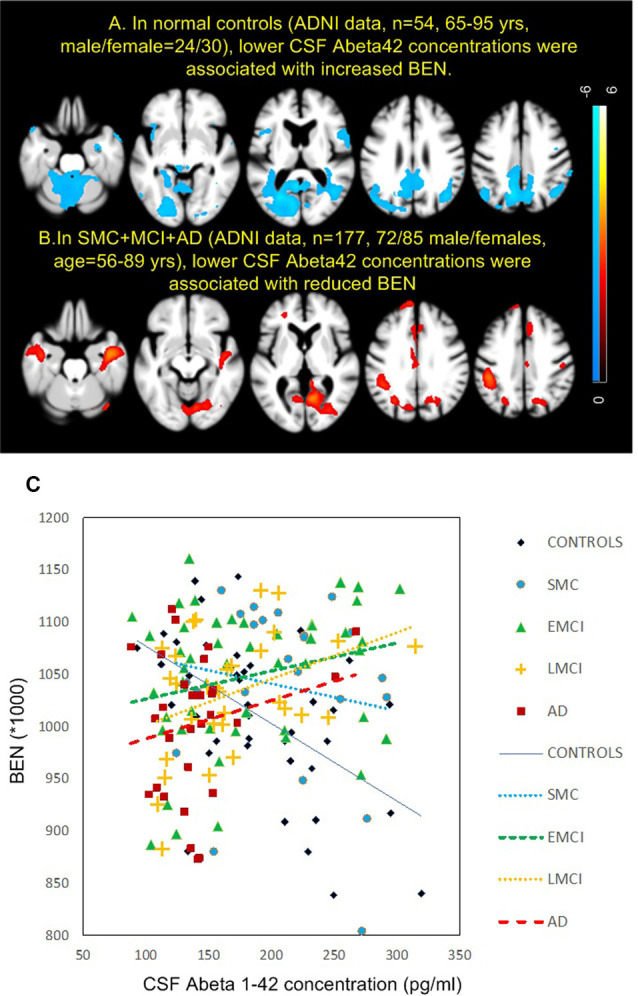
Brain entropy (BEN) vs. cerebrospinal fluid (CSF) Aβ associations in panel **(A)** controls and **(B)** MCI and AD. **(C)** The associations for each sub-group in precuneus. The red cross in the 4th image in panel **(A)** indicates the location of the precuneus region of interest. The lines in panel **(C)** depict the linearly fitted associations. EMCI, early mild cognitive impairment; LMCI, late mild cognitive impairment.

Figure 4 shows the results of BEN vs. AD pathology association analyses. AD pathology was measured by CSF Aβ_1–42_ peptide concentration with lower CSF Aβ meaning higher brain Aβ depositions. Controls and patients showed opposite BEN vs. CSF Aβ associations in nearly the same brain regions. As CSF Aβ_1–42_ is inversely related to beta amyloid depositions in the brain (Grimmer et al., 2009), the negative CSF Aβ-BEN correlation found in controls (Figure 4A) means BEN in DMN, MTL, lateral PFC, and visual cortex may increase with brain beta amyloid depositions. In patients (Figure 4B), BEN decreases with brain beta amyloid depositions. Figure 4C shows the scatter plots for all subgroups. Controls and SMC showed opposite BEN vs. CSF Aβ relationship though the correlation was statistically significant only in controls (*r*^2^ = 0.282, *p* = 4.3e−4) and LMCI (*r*^2^ = 0.14, *p* = 0.04). Similar BEN vs. AD pathology associations were found when we used tau/Aβ ratio or p-tau/Aβ ratio as the pathology indicator.

**Figure 5:**
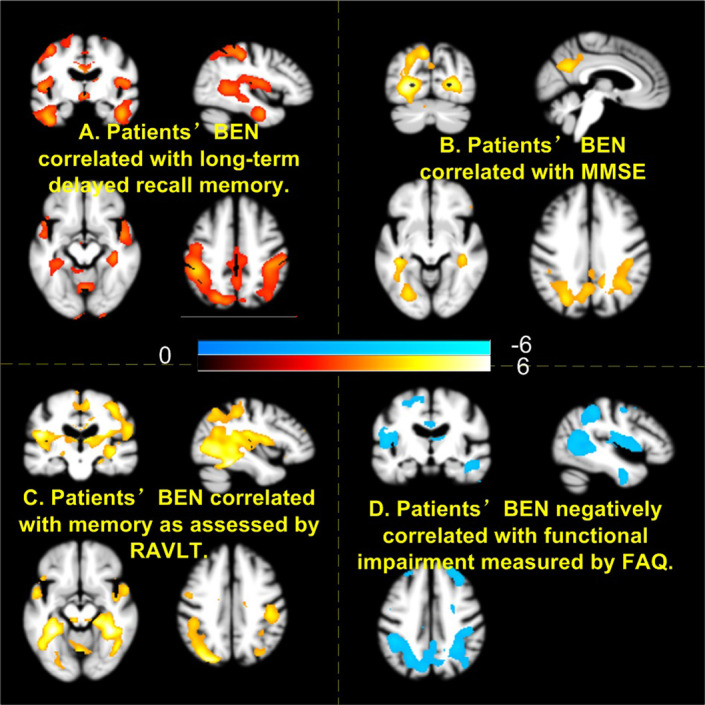
Higher brain entropy (BEN) in parietal cortex, temporal cortex associated with more severe impairment of memory **(A,C)**, cognitive **(B)**, and daily functions **(D)**. Red and cool colors mean positive and negative correlations, respectively. MMSE, Mini-Mental State Examination; RAVLT, Rey Auditory Verbal Learning Test; FAQ, Functional Activity Questionnaire.

Figure 5 shows the associations of BEN to cognitive and daily functional impairment for the composite patient group. Age, sex, and education years were regressed out. Both Figure 5A (delayed-recall) and Figure 5C (RAVLT) show a positive correlation of BEN to memory function, meaning that a lower BEN in the elucidated regions (DMN, MTL) corresponds to a more severe memory deficit. BEN in DMN and hippocampus was positively related to MMSE (Figure 5B), suggesting patients with more cognitive impairments have lower BEN. Lower BEN in DMN, temporal cortex, and PFC was further related to more severe daily functional disability as measured by FAQ.

## Discussion

We assessed resting state BEN as a proxy for assessing the latent brain reserve and proposed a heuristic inverse-U shape BEN model to explain the aging-related functional brain alterations and the pathology vs. AD symptom discrepancy. The validity of BEN as a reserve proxy was examined by the BEN vs. age, education, and cognitive performance association studies. The inverse-U shape model was evaluated by comparing BEN across normal aging and patients with different stages of disease in the AD continuum as well as by the neurobehavioral and pathological association analyses. The major findings are as follows: (1) the cross-sectional analysis demonstrated that BEN first slightly increased from normal aging to SMC and to EMCI, but quickly fell below the BEN level of normal controls in LMCI, and reduced further in AD with an accelerated pace; (2) BEN presented different age and education effects in normal aging and AD continuum. It increases with age in normal aging but decreases with age in the AD continuum. It decreases with education years in normal aging, but is not correlated with education any more in the AD continuum; (3) BEN showed totally opposite associations with CSF Aβ depositions. The BEN vs. CSF Aβ correlation was negative in normal aging but became positive in the AD continuum; and (4) low BEN was correlated with more severe cognitive impairment and daily function disability in the AD continuum. These findings highlighted resting BEN in DMN, MTL, and PFC, which have been implicated in different neuroimaging-based aging and AD studies (Ries et al., 2008; Ouchi and Kikuchi, 2012; Weiner et al., 2013; Wang, 2016; Badhwar et al., 2017; Anthony and Lin, 2018).

These findings proved the hypothetical inverse-U shape BEN model as depicted in Figure 1: resting BEN changed from cognitively normal controls to AD following an apparent inverse-U shape; BEN increases with age and pathological depositions but decreases with longer education years in normal aging; in the AD continuum, BEN decreases with age and is not correlated with education anymore as the reserve-based function compensations fail. Age had deleterious effects on BEN (BEN increases with age), but the effects were surpassed by a potential overaction of brain reserve after clinical observable memory or cognitive problems emerged. Controls and patients showed opposite age effects on BEN, which can be explained by the substantially reduced BEN in LMCI and AD. The lack of education effects in patients may suggest a failure of the compensation role of BEN especially in later stages of dementia. Education years showed effects of reducing BEN in the cognitively normal elderly, but the effects diminished in the AD continuum, indicating a weakening or failure of the reserve compensation as suggested by the brain reserve literature (Stern, 2006, 2012; Stern et al., 2018). This compensation weakening or failure was further supported by the BEN vs. behavior correlations showing that lower BEN in DMN/MTL/PFC is correlated to more severe cognitive impairment and daily functional disability. The opposite BEN evolution processes in normal controls and the disease continuum were supported by the AD pathology association findings: higher AD pathology deposition (reflected by lower CSF Abeta level) is associated with increased BEN in the cognitively normal elderly, suggesting an AD pathology–related functional deterioration in normal aging. In contrast, the pathology–BEN association was switched to the opposite in the AD continuum showing more brain pathology corresponding to lower DMN/MTL/PFC BEN. This pathology-related BEN reduction (rather than increase) indicates an escalated demand of compensation triggering a reserve overaction, which unfortunately cannot be achieved anymore. The dramatically reduced BEN eventually leads to accelerated functional impairments as the brain activity still needs a certain level of entropy to keep its functional flexibility (Tagliazucchi et al., 2012; Haimovici et al., 2013). Low entropy may also indicate a low energy state, which is supported by the well-known hypo-perfusion/hypo-metabolism state found in AD (Johnson et al., 2005; Ruitenberg et al., 2005; Chao et al., 2009; Hu et al., 2010; Chen et al., 2011; Musiek et al., 2012; Wang et al., 2013; Liu et al., 2014; Wang, 2014; Verclytte et al., 2016; Daulatzai, 2017).

The BEN variation patterns from normal aging to the AD continuum are consistent with our initial finding reported in 2016 (Li and Wang, 2016) and the AD hypo-entropy literature (Stam et al., 2003; Jeong, 2004; Abásolo et al., 2006; Gómez and Hornero, 2010; Mizuno et al., 2010; Yang et al., 2013; Wang et al., 2017). Different from these previous studies, the current study provided more comprehensive data regarding the change patterns of BEN across different disease stages, the associations of BEN to AD pathology, the associations with age and education, and the link to clinical consequences. The link of BEN to brain reserve was examined through its correlation to education years, which is a widely used index of cognitive reserve. The BEN–brain reserve association was also evidenced by the correlation to neurobehaviors in the patients. Although we did not find a significant correlation between BEN and neurobehavior measures in the healthy elderly controls, we observed significant negative correlation between BEN and cognitive function and education years but significant positive correlation between BEN and age in 866 young healthy adults in a article under peer review. Those data suggest that BEN vs. neurobehavior correlation in elderly controls may still exist but require a larger sample size to be identified.

Brain reserve was proposed to explain the individual difference of tolerating the pathology-induced functional alterations (Stern, 2006, 2012; Stern et al., 2018). Because brain reserve is non-specific to any overt brain function, the null-hypothesis resting state activity which play a role in function facilitation (Raichle et al., 2001; Raichle and Gusnard, 2002; Raichle, 2006; Pizoli et al., 2011) has been postulated to be related to brain reserve in several studies (Arenaza-Urquijo et al., 2013; Bozzali et al., 2015; Marques et al., 2016; Franzmeier et al., 2017; Li et al., 2020). Different from these previous studies, the current study focused on regional resting brain activity, which may either be the action or the outcome of brain reserve facilitation or compensation. We chose entropy as the proxy to characterize the neural substrates of brain reserve because any system including human brain is prone to errors and deteriorations which inevitably leads to entropy increase (Finch et al., 2000; Hayflick, 2004, 2007a,b; Drachman, 2006). Without compensation, brain activity will be disrupted and provide no function. No matter how functional compensation by brain reserve works (which is unknown), the compensation outcome should be a reduction of entropy. Another rationale for choosing BEN is that BEN is inversely related to coherence (low BEN means high coherence) and brain activity coherence has been shown to be fundamental to high-order brain functions such as memory, attention, perception, and coordination (Pesaran et al., 2002, 2008; Buzsáki and Draguhn, 2004; Buzsaki, 2006; Womelsdorf et al., 2006; Buschman and Miller, 2007; Gregoriou et al., 2009; Schroeder and Lakatos, 2009; Siegel et al., 2009; Saleh et al., 2010; Hagan et al., 2011; Buzsáki and Watson, 2012; Dean et al., 2012; Henry and Obleser, 2012; Lega et al., 2012; Salazar et al., 2012; Thut et al., 2012; Rigotti et al., 2013; Calderone et al., 2014; Hawellek et al., 2016; Wong et al., 2016). Loss of temporal coherence interrupts inter-neuronal then inter-regional communications. Restoring brain coherence can therefore fix the related brain dysfunctions. For example, a recent study showed that enhancing coherence improved memory for older people (Reinhart and Nguyen, 2019). However, too much coherence (very low BEN), such as in the sedation or coma state (Viertiö-Oja et al., 2004; Perez et al., 2019), will make the brain too rigid, unable to form variable brain activity patterns. This situation can happen in the AD continuum because of the escalated compensation demand from the progressive brain function deterioration caused by both aging and AD pathology.

Limitations exist in this study. First, these findings were based on cross-sectional data and must be further confirmed with longitudinal data. Second, the negative BEN vs. education correlations seem to be contradictory to a previous large size study showing positive correlations between BEN and intelligence (Saxe et al., 2018). We have to note that the suprathreshold regions between this study and Saxe et al. (2018) did not overlap with ours mainly in the parietal cortex and theirs in inferior frontal and temporal regions and cerebellum. In an independent study based on 862 young healthy adults’ high-resolution, high signal-to-noise-ratio long rsfMRI data from the Human Connectome Project, we observed the same negative education vs. BEN correlations in parietal cortex as well as prefrontal cortex (Wang, 2020a,b). The consistent findings across two different cohorts with different age ranges and different imaging acquisition parameters prove the rigor of the negative BEN vs. education findings. Third, although the heuristic BEN model predicts a reserve compensation-related BEN reduction, the rsfMRI-derived BEN represents the sum of the aging-related BEN and the compensation-induced BEN reduction and we cannot separate them. In other words, we cannot assess the compensation-related BEN reduction independently. A fifth concern is the physiological noise such as motion, cardiac, and respiratory pulsations. Although we followed the standard processing steps for motion correction, residual motion effects removal, and physiological noise filtering, residual effects may still exist. As those confounds are unlikely correlated with all the assessed variables such as age, education, pathology, and cognitive measures, the major BEN effects identified in this article should be still related to neuronal events. Finally, BOLD signal is also contributed by vascular effects. Because vascular abnormality is a known risk factor of AD, vascular contributions to BOLD fMRI signal may be even larger than in healthy controls. Therefore, the observed resting BOLD fMRI-derived BEN effects likely contained both neuronal and vascular effects too.

In summary, rsfMRI-derived BEN provides a potential proxy to assess the brain circuits underlying brain reserve; BEN follows an inverse-U curve when normal aging progresses into AD. The heuristic BEN progression model may provide a potential tool for early detection of AD and disease modification development given the recent evidence of that resting BEN can be modulated using non-invasive transcranial magnetic resonance stimulation (Chang et al., 2018; Song et al., 2018).

## Data Availability Statement

The datasets presented in this study can be found in online repositories. The names of the repository/repositories and accession number(s) can be found below: http://adni.loni.usc.edu/.

## Ethics Statement

The studies involving human participants were reviewed and approved by IRB of University of Maryland, Baltimore, MA, USA. The patients/participants provided their written informed consent to participate in this study.

## Author Contributions

The author confirms being the sole contributor of this work and has approved it for publication.

## Conflict of Interest

The author declares that the research was conducted in the absence of any commercial or financial relationships that could be construed as a potential conflict of interest.

## Footnotes

^1^http://www.adni-info.org

^2^https://www.fil.ion.ucl.ac.uk/spm/
